# What Belongs Together Retrieves Together – The Role of Perceptual Grouping in Stimulus-Response Binding and Retrieval

**DOI:** 10.5334/joc.217

**Published:** 2022-04-12

**Authors:** Philip Schmalbrock, Andrea Kiesel, Christian Frings

**Affiliations:** 1 Department of Psychology, University of Trier, DE; 2 Albert-Ludwigs-University, Freiburg, DE

**Keywords:** S-R binding, perceptual grouping, gestalt principles

## Abstract

Nowadays there is consensus that stimulus and response features are partially represented in the same coding format furthering the binding of these features into event files. If some or all features comprised in an event file repeat later, the whole file can be retrieved thereby modulating ongoing performance (leading to so-called stimulus-response binding effects). Stimulus-response binding effects are usually investigated in sequential priming paradigms where it is assumed that binding occurs in the prime and retrieval in the probe. Importantly, binding and retrieval are not exclusive for targets but also apply to distractor stimuli. A previous study showed that distractor-binding effects were affected by perceptual grouping: Binding effects were significantly larger when stimuli were grouped compared to ungrouped stimuli. Recent theorizing suggests that binding and retrieval are two separate processes that can be individually modulated. Against this background, it is not possible to pinpoint the modulating influence of perceptual grouping on either process at this point in time. Therefore, we adapted the previous study design in two experiments to observe the effect of perceptual grouping on both processes in isolation. Results indicate that perceptual grouping did not impact binding but retrieval: Distractor-response retrieval was reduced when target and distractor were presented in separate objects. Our results thus support recent theorizing on the separation of binding and retrieval.

## Introduction

Even executing the simplest action activates the complex machinery in our brain to perform the task effortlessly and often without a second thought. Take, for example, the simple act of grabbing a bottle of water. Although trivial in its consequences, this action requires several processes that culminate in holding the bottle. These processes must range from perceiving and processing the bottle to planning, executing and correcting the grabbing action. One line of research that is concerned with this stream of processes is the action control literature.

The *Theory of Event Coding* (TEC, Hommel et al., 2001) assumes that perception and action actually refer to the same kind of sensorimotor activity (see also Hommel, 2009). That is, perception and action both require a systematic movement of one’s body to elicit sensory information from the environment (Hommel, 2022). Actions are assumed to be represented as their anticipated perceptual effects – an assumption in line with the ideo-motor framework (see Shin et al., 2010, for a review). Thus, stimulus and response features can both be represented in a common representational format (*common coding* assumption, Prinz, 1997) which enables us to represent stimulus and response features in one coherent unit, termed *event file* (Hommel, 2004, see also Logan, 1988 for a similar concept). Stimulus and response features are bound or integrated (we will refer to this process as *binding*) into an event file so that information that belongs together cannot be intermixed with rivaling information or event files (Stoet & Hommel, 1999). This is beneficial to behavior because features that belong to one event file cannot be part of another and thus cannot lead to redundant or mutually excluding representations of the same action (*code occupation*, Prinz, 1997). It has been suggested that these binding mechanisms are strongly intertwined with working memory (e.g., Hommel et al., 2001; Logan, 1988) or might occur in working memory (Phaf & Wolters, 1997; Singh & Schubert, 2021; Treisman & Zhang, 2006; Wolters & Raffone, 2001).

Importantly, event files do not decay completely for a short period after their creation (Frings & Hommel, 2020). Thus, when some or all features comprised in an event file repeat in a later episode, the whole event file can be *retrieved* (Hommel, 1998). That is, retrieved event files can actually improve performance (faster reaction times, less errors) when all previous features repeat (compared to a full change condition). These repetition benefits happen because the previous response can be reused without the additional processing steps that are needed to compute a new response (Henson et al., 2014). However, when only some features repeat at a later point in time, performance is hampered compared to a full change or full repetition condition (longer reaction times, more errors). These partial repetition costs have been attributed to a competition between the previous response contained in the event file and the demands of the present episode (*horserace account*, Frings & Moeller, 2012). This conflict has to be resolved and thus performance delay increases due to additional, time-consuming processes (e.g., Geissler et al., 2021; code confusion at retrieval was also proposed as an explanation, Fournier et al., 2020; Mattson et al., 2012; Mattson & Fournier, 2008); such costs and benefits are often referred to as S-R *binding effects*.

A recent adaptation of TEC, the *Binding and Retrieval in Action Control* framework (BRAC; Frings et al., 2020) emphasizes that binding and retrieval are two separate processes that both contribute to S-R binding effects. In addition, it was suggested that each process can be modulated by top-down and bottom-up influences and that modulations must not necessarily target binding and retrieval processes equally. For example, Laub et al. (2018) found that presenting stimuli in similar colors enhanced S-R binding while presenting stimuli in dissimilar colors enhanced retrieval. Or Hommel et al. (2014) reported that cuing a feature dimension affected only retrieval but not binding. Several other publications also point to the necessity to distinguish between binding and retrieval processes (Hommel et al., 2014; Memelink & Hommel, 2013; Mocke et al., 2020; Schmalbrock et al., 2021; Schmalbrock & Frings, Manuscript submitted for publication, 2021). However, the studies by Laub et al. (2018) and Hommel et al. (2014) highlight modulators can target binding and retrieval independently from each other and may have different impacts on both processes. Perceptual grouping has been established as one modulator of distractor-based S-R binding effects *in general* (Frings & Rothermund, 2011). Yet, this previous study did not distinguish between the actual processes, binding and/or retrieval, being modulated. We were, therefore, interested in determining the role perceptual grouping plays for binding/retrieval separately.

### The influence of perceptual grouping on binding versus retrieval

Perceptual grouping describes one of the earliest investigated *gestalt principles* (e.g., Wertheimer, 1923) – different sets of principles that determine the perceived compositional configuration of our perception (see Wagemans et al., 2012 for a review). Specifically, perceptual grouping comprises several principles that can lead to the impression that certain features belong more together than others. This early research showed that proximity of stimuli, similarity of color, size and orientation, as well as common fate (Wertheimer, 1923) and the later added principle of common region (Palmer, 1992) determine whether we perceive stimuli or features of a stimulus as belonging together or not. These principles were further extended and now also include synchrony (e.g., Alais et al., 1998) and elemental connectedness (Palmer & Beck, 2007) – see Wagemans et al. (2012) for an overview of known perceptual grouping mechanisms.

Previous research investigated the modulating influence of perceptual grouping on S-R binding in the context of the distractor-response binding paradigm (DRB, Frings et al., 2007). DRB is a sequential priming paradigm where participants respond to two consecutive displays (the prime display followed by the probe display). Participants make a discriminatory response towards the target while distractors are present, in both prime (binding) and probe (retrieval). Importantly, responses and distractors either repeat or change independently from prime to probe, thus, resulting in full repetition trials (same response and distractor in prime and probe), full change trials (different response and distractor in prime and probe) and partial repetition trials (same response but different distractor in prime and probe, or vice versa). Intriguingly, depending on the trial type, probe performance differs. In full repetition trials, performance improves compared to full change trials (faster response time, fewer errors) because the previous event file is retrieved, including the previous response, which enables the cognitive system to reuse the previous response without additional computations. In partial repetition trials, however, probe performance decreases compared to full change or full repetition trials (slower response time, more errors) because the previous event file conflicts with the demands of the present episode. In full change trials, performance is often better than in partial repetition trials but slightly worse than in full repetition trials.^1^ These effects were labeled *distractor-response binding* effects (DRB effects) to stress that the stimulus that is bound and retrieves is not a target but a distractor (Frings et al., 2007).

Using the DRB paradigm, Frings and Rothermund (2011) introduced several different manipulations of perceptual grouping principles. Through the principle of similar orientation, symmetry and common region they manipulated whether distractor and target were perceived as belonging together or as separate units. Intriguingly, in this study, DRB effects almost always emerged only with stimuli that belong together (grouped) but were reduced/eliminated with separated stimuli (ungrouped). Against the background of the BRAC framework, it seems important to ask whether grouping modulates binding of features into event files or their retrieval.

### The Present Study

We adapted and simplified the design of Experiment 6 from Frings and Rothermund (2011) to determine the relationship between perceptual grouping and binding versus retrieval. We reduced stimulus complexity to just two items, a target letter and a distractor number. Participants responded to the identity of a target letter (by pressing the F or J key) and were instructed to ignore the distractor. Further, we also introduced boxes as a means to manipulate perceived grouping. That is, target and distractor could be framed by the same box or both stimuli were presented in separate boxes (with constant eccentricity between targets and distractors in all conditions). Crucially, we only applied the grouping manipulation to prime *or* probe.

**Figure 1:**
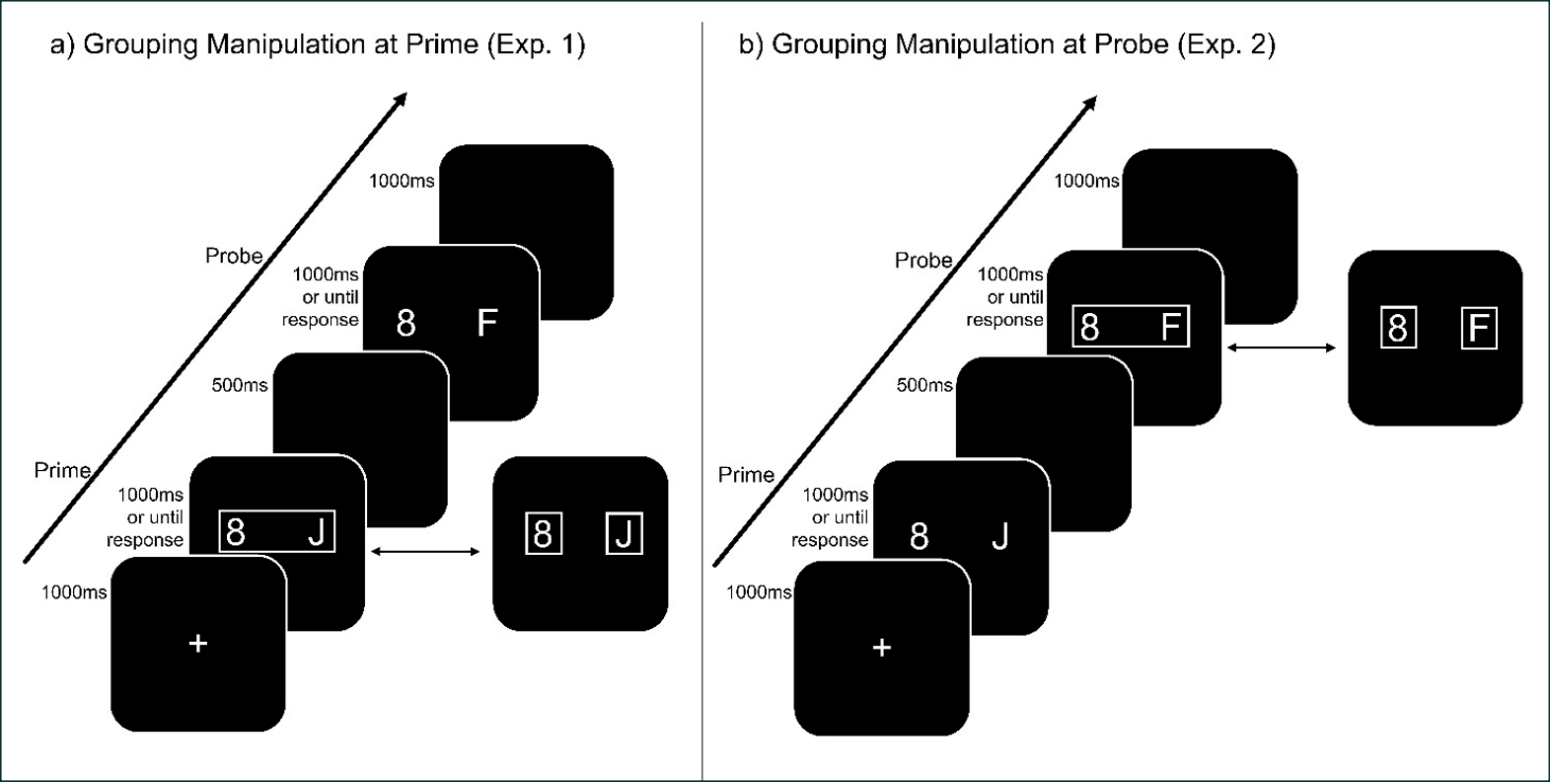
**Exemplary experimental trial.** Distractor repetition with response change trial. **a)** Experiment 1, prime manipulation. **b)** Experiment 2, probe manipulation. Responses were made towards the letters identity. Perceptual grouping was manipulated by presenting target and distractor within a single box or in two separate boxes. Note that stimuli are not drawn to scale.

In Experiment 1, we manipulated grouping only in the prime (i.e., at binding). In each trial, prime target and distractor are presented in one or two separate boxes. In the probe, however, the target and distractor are presented without any box framing (see ***Figure 1a***). Thus, stimuli are only (un-) grouped at binding while the level of grouping in the probe is constant over all trials. If the observed impact of grouping on binding effects were due to modulation of binding at the prime, we should observe smaller binding effects (or no binding effects at all) when target and distractor were ungrouped.

In Experiment 2, we manipulated grouping only in the probe (i.e., at retrieval). That is, the target and distractor were either framed in one or two boxes in the probe while no boxes were shown in the prime (see ***Figure 1b***). If the observed impact of grouping on binding effects were due to modulation of retrieval at the probe, we should observe smaller binding effects (or no binding effects at all) when target and distractor were ungrouped.

## Experiment 1 (Perceptual Grouping at Prime)

### Methods

#### Participants

90^2^ students of Trier University (60 female and 30 male; 77 right-handed and 13 left-handed) with a median age of 22 years (range = 20 to 36 years) participated. They consented via an online form and received partial course credit for finishing the experiment. This study followed the ethical standards defined by Trier University. The sample size was calculated according to the previous studies by Frings and Rothermund (2011) investigating modulating effects of perceptual grouping on S-R binding, which led to small-sized effects (*d_z_* = 0.30). Thus, we planned to run *N* = 90 participants, leading to a power of 1 – *β* = 0.80 (assuming an alpha = 0.05; GPower 3.1.9.2; Faul et al., 2007).

#### Design

For Experiment 1, three within-participant factors were varied: Response relation (response repetition vs. response change), distractor relation (distractor repetition vs. distractor change), and perceptual grouping (grouped vs. ungrouped) in the prime display.

#### Apparatus & Stimuli

Participants were redirected from Trier University’s recruitment platform (*SONA Systems*) to the experimental platform ‘Pavlovia’ (*pavlovia.org*; Peirce & MacAskill, 2018). The experiment was programmed in Psychopy (Peirce et al., 2019, Version 03.01.2020).

Two distinct displays were presented (see ***Figure 1***): prime and probe display. In the prime and probe displays two white symbols (RGB_255_ = 255, 255, 255) were shown, a letter (target; J or F) and a number (distractor; randomly drawn from 1, 3, 4, 8), each with a font size of 25 pixels and a distance of 25 pixels left and right from screen center. Importantly, in grouped trials, a white box (Width × Height: 90 × 40 pixels; RGB_255_ = 255, 255, 255) surrounded prime target and distractor. In ungrouped trials, each prime symbol was surrounded by a separate box (each 40 × 40 pixels; RGB_255_ = 255, 255, 255). No boxes were shown in the probe. All stimuli were presented in front of a black (RGB_255_ = 0, 0, 0) background. Positions for prime target and distractor were randomly sampled from the two available positions. However, target and distractor positions always repeated from prime to probe.

#### Procedure

Instructions were presented to the participants via text on the screen. Participants were instructed to place the left index finger on the ‘F’-key and the right index finger on the ‘J’-key and to identify the letter via a key press as fast and as accurately as possible while ignoring the number stimulus. A training block with 20 trials was completed before the experimental block started. Here, participants received positive (“Richtig!”, English: “Correct!”) and negative (“Falsch!”, English: “Wrong!”) performance feedback after each prime and probe response, respectively. In the experimental block, feedback was only given directly after a wrong response was given in either prime or probe respectively. The trial was not terminated after an incorrect response. The experimental task consisted of two consecutive responses towards a target in the prime display and then to a target in the probe display.

The experimental block consisted of 256 trials with a self-paced break after each 32-trial block. Thus, a single trial consisted of the following chain of events: A fixation mark (+) was shown for 1000 ms at the screen center. Then, the prime display was presented for up to 1000 ms or until a response was registered. After the response, a blank display was shown for 500 ms. Then the probe display was presented, also for 1000 ms or until a response was registered. Each trial was separated from the next by a blank display presented for 1000 ms.

Three factors were varied orthogonally: response relation, distractor relation, and perceptual grouping in the prime. In response repetition trials, the same response required in the prime was required in the probe. In contrast, in response change trials, a different response was required in prime and probe. In distractor repetition trials, the prime distractor identity was repeated as the probe distractor. In distractor change trials, the probe distractor identity differed from the prime distractor identity. In grouped trials, prime target and distractor were surrounded by a single box. In ungrouped trials, prime target and distractor were each surrounded by a separate box. The perceptual grouping manipulation was varied trialwise.

### Results

Data processing and analysis were done with R (R Core Team, 2019; R version 3.6.1). The package ‘dplyr’ (Wickham et al., 2019) was used for data processing and aggregation.

The distractor-response binding effect is computed as the distractor repetition benefit in response repetition trials minus the distractor repetition interference in response change trials ([RRDC-RRDR]-[RCDC-RCDR]).^3^ DRB effects were compared using *t*-tests complemented by Bayesian *t*-tests (Rouder et al., 2009) whose Bayes factor (*BF*_01_) quantify the evidence in favor of the null hypothesis relative to the evidence in favor of the alternative hypothesis (see the **Supplementary Material** for the analysis via ANOVA). Values between 1 and 3 indicate weak/anecdotal evidence in favor of the null hypothesis, and values >3 indicate positive/strong evidence for the null hypothesis. In contrast, values from 1 to .33 indicate weak/anecdotal evidence for the alternative hypothesis, and values < .33 indicate positive/substantial evidence for the alternative hypothesis (Jarosz & Wiley, 2014). Bayes factors were computed using the package ‘BayesFactor’ (Morey & Rouder, 2018).

#### Data Processing

For the analysis of probe performance, only probe trials with correct answers in the prime were considered for error and reaction time (RT) analysis. Additionally, only trials with RT longer than 200 ms and shorter than 1.5 interquartile ranges over the third quartile of each person’s RT distribution were analyzed (see Tukey, 1977). That is, 10% of all trials were excluded due to these constraints. For the analysis of probe RT, all trials with wrong probe responses were also excluded, that is an additional 4% of all trials (14% in total; range: 5% to 71%). For the analysis of prime performance, the same (where applicable) criteria were applied.

#### Analysis of Prime Performance

A paired *t*-test revealed a significant difference in prime RT for grouped prime displays (*M* = 538 ms, *SD* = 57) compared to ungrouped prime displays (*M* = 540 ms, *SD* = 56), one-sided *t*(89) = 2.62, *p* = .005, *d_z_* = 0.27, *BF*_01_ = 0.18. The same analysis on prime error rate revealed no difference between grouped prime displays (*M* = 6%, *SD* = 5) and ungrouped prime displays (*M* = 6%, *SD* = 5), one-sided *t*(89) = 0.28, *p* = .390, *d_z_* = 0.03, *BF*_01_ = 6.79.

#### Probe Reaction Times

**Figure 2:**
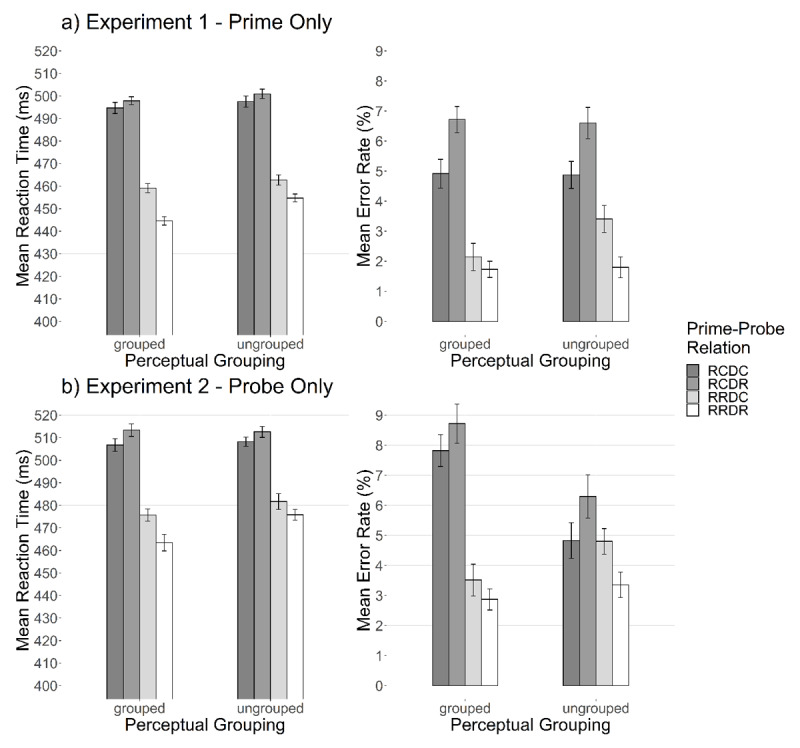
**Mean Performance for Experiment 1 and 2.** Reaction times (left panels) and error rates (right panels) for a) Experiment 1 and b) Experiment 2. The prime-probe relation reflects the interaction between response relation (repetition vs. change) and distractor relation (repetition vs. change). Response repetition/change trials are abbreviated with RR/RC. Distractor repetition/change trials are abbreviated with DR/DC. Thus, this leads to the four trial types response repetition with distractor repetition (RRDR), response repetition with distractor change (RRDC), response change with distractor repetition (RCDR), and response change with distractor change (RCDC). Note that performance should be impaired in partial repetition trials (RRDC and RCDR) compared to complete repetition or complete change trials (RRDR and RCDC). Error bars indicate within-participant error of the mean (Morey, 2008).

A paired *t*-test revealed that DRB effects in the grouped condition (*M* = 18 ms, *SD* = 36) were not significantly larger than the ungrouped condition (*M* = 11 ms, *SD* = 35), *t*(89) = –1.06, *p* = .147, *d_z_* = 0.11, *BF*_01_ = 2.95 (one-sided; see ***Figure 2*** and **Appendix A**). Post-Hoc analysis evidenced that the binding effect was significantly different from zero for grouped prime trials (one-sided, *t*(89) = 4.70, *p* <.001, *d_z_* = 0.50; *BF*_01_ < 0.01), and for ungrouped prime trials (one-sided, *t*(89) = 3.05, *p* <.003, *d_z_* = 0.32; *BF*_01_ = 0.06).

#### Probe Error Rates

A paired *t*-test revealed that binding effects for grouped prime trials (*M* = 2%, *SD* = 8) were not significantly larger than ungrouped prime trials (*M* = 3%, *SD* = 8), one-sided *t*(89) = 0.938, *p* = .351, *d_z_* = 0.10; *BF*_01_ = 15.68. Post-Hoc analysis evidenced that the binding effect was significantly different from zero for ungrouped prime trials (one-sided *t*(89) = 4.10, *p* < .001, *d_z_* = 0.32; *BF*_01_ < 0.01), and for grouped prime trials (one-sided *t*(89) = 2.49, *p* = .015, *d_z_* = 0.26; *BF*_01_ = 0.24).

### Discussion

In Experiment 1, we tested whether perceptual grouping affected distractor-response binding. That is, prime stimuli were either presented in the same box (grouped) or in two separate boxes (ungrouped). Previous work investigated perceptual grouping in DRB *simultaneously* (Frings & Rothermund, 2011). They found no DRB effects for ungrouped stimuli and suggested that this might be due to the impact grouping has on binding. Our results stand in stark contrast to these findings as we did not find a modulating influence on binding (and the Bayes factor on RTs indicated weak/anecdotal evidence in favor for the null hypotheses while the Bayes factor on error rates revealed clear evidence in favor for the null). Thus, the modulating influence of perceptual grouping might target retrieval and not binding. The significant impact of our grouping manipulation on prime performance reveals that our manipulation worked but did not affect binding. This was somewhat expected as findings from the gestalt literature suggest that participants generally show improved performance if stimuli are grouped compared to ungrouped stimuli (Beck & Palmer, 2002; Palmer & Beck, 2007). Our second experiment thus investigated the relationship between distractor-response retrieval and perceptual grouping.

## Experiment 2 (Perceptual Grouping at Probe)

### Methods

#### Participants

90 students of Trier University participated (66 female and 24 male; 88 right-handed and 2 left-handed) with a median age of 22 years (range = 18 to 37 years). Participants consented via an online form and received partial course credit for finishing the experiment. This study followed the ethical standards defined by Trier University. The sample size was determined in the same way as for Experiment 1.^4^

#### Design

For Experiment 2, three within-participant factors were varied: Response relation (response repetition vs. response change), distractor relation (distractor repetition vs. distractor change), and perceptual grouping (grouped vs. ungrouped) in the probe display.

#### Apparatus & Stimuli

Stimuli and apparatus were identical to Experiment 1.

#### Procedure

The procedure of Experiment 2 was identical to Experiment 1 with the important difference that the perceptual grouping manipulation took place in the probe (see ***Figure 1b***).

### Results

#### Data Processing

14% of all trials were excluded due to the same cut-off criteria as in Experiment 1. Additionally, 5% (adding to 19% in total; range: 4% to 89%) of all trials were excluded due to wrong probe responses.

#### Analysis of Prime Performance

A paired *t*-test revealed no significant difference in prime RT for grouped prime displays (*M* = 526 ms, *SD* = 51) compared to ungrouped prime displays (*M* = 525 ms, *SD* = 48), one-sided *t*(89) = –0.43, *p* = .666, *d_z_* = 0.05, *BF*_01_ = 11.67. The same analysis on prime error rate revealed no difference between grouped prime displays (*M* = 7%, *SD* = 8) and ungrouped prime displays (*M* = 7%, *SD* = 7), one-sided *t*(89) = 0.45, *p* = .327, *d_z_* = 0.05, *BF*_01_ = 5.80.

#### Probe Reaction Times

A paired *t*-test revealed that binding effects in the grouped condition (*M* = 19 ms, *SD* = 36) were larger than in the ungrouped condition (*M* = 10 ms, *SD* = 32), *t*(89) = –1.81, *p* = .036, *d_z_* = 0.19, *BF*_01_ = 0.92 (one-sided; see ***Figure 2*** and **Appendix A**). Post-Hoc analysis evidenced that the binding effect was significantly different from zero for grouped probe trials (one-sided, *t*(89) = 4.92, *p* <.001, *d_z_* = 0.52; *BF*_01_ < 0.01), and for ungrouped probe trials (one-sided, *t*(89) = 3.07, *p* < .001, *d_z_* = 0.23; *BF*_01_ = 0.06).

#### Probe Error Rates

For the same analysis on error rates, only trials with correct prime responses but incorrect probe responses were considered (i.e., 5% of all trials were relevant for this analysis).

A paired *t*-test revealed that binding effects for grouped prime trials (*M* = 2%, *SD* = 8) were not significantly larger than ungrouped prime trials (*M* = 3%, *SD* = 12), one-sided *t*(89) = 0.93, *p* = .355, *d* = 0.10; *BF*_01_ = 15.61. Post-Hoc analysis evidenced that the binding effect was not significantly different from zero for grouped probe trials (one-sided, *t*(89) = 1.86, *p* = .066, *d_z_* = 0.20; *BF*_01_ = 0.86), and not for ungrouped probe trials (one-sided, *t*(89) = 2,35, *p* = .021, *d_z_* = 0.25; *BF*_01_ = 0.33).

## General Discussion

The present study investigated the role of perceptual grouping for DRB effects. A previous study (Frings & Rothermund, 2011) showed a modulating influence of grouping on DRB effects in general but did not allow to differentiate between modulation of binding and/or retrieval. Against the background of recent theorizing (the BRAC framework; Frings et al., 2020) we investigated whether grouping modulates event file binding and event file retrieval.

In two separate experiments, we introduced perceptual grouping only to the prime or probe display of a standard DRB paradigm.^5^ Since the manipulation was only applied to one but not the other display, the resulting data may show possible effects of grouping on event file binding irrespective of event file retrieval or vice versa (note however the limitations discussed below). DRB effects were modulated by grouping in the second experiment, that is, our data suggest that perceptual grouping exerts its modulating influence on retrieval (irrespective of binding processes).

However, it is important to note that the effect on retrieval is rather small (*d_z_* =.19). In that regard, it is very similar to the effect from the previous study by Frings and Rothermund (2011; *d_z_* = .27). This difference is possibly caused by how we implemented our manipulation (which could not be helped). That is, we possibly weakened the reliability of our effect through the encoding specificity of S-R binding effects (Laub & Frings, 2020). Specifically, encoding specificity in sequential priming paradigms refers to the idea that retrieval is diminished if prime and probe displays are dissimilar. In the present experiments, the defining feature of the manipulated display (i.e., the boxes) was absent in the other display. Thus, the similarity between prime and probe displays in our study was reduced if compared to the study of Frings and Rothermund (2011) which might explain why we observed somewhat weaker binding effects in general. This is also the reason why locations are always repeated from prime to probe displays. Due to encoding specificity, location changes would have weakened the S-R binding effects even further. Even worse, location seems to be rather special for S-R binding processes and location changes (especially between prime and probe) induce different forms of binding (Singh & Frings, 2020).

In addition, the prime target location was always unpredictable, while the probe target location was always predictable (by the prime target location). Prime target location unpredictability should lead to an additional visual search element in the prime task. That is, participants probably have to locate the target first before they can actually respond to it. Assuming that participants always use the location information in the probe, participants should be slower to respond in the prime than in the probe – which they were (Exp. 1: Prime: *M* = 539 ms, *SD* = 54, Probe: *M* = 475 ms, *SD* = 63, *t*(89) = –10.45, *p* < .001, *d_z_* = 1.10, *BF*_01_ < 0.01; Exp. 2: Prime: *M* = 525 ms, *SD* = 49, Probe: *M* = 492 ms, *SD* = 84, *t*(89) = 3.88, *p* < .001, *d_z_* = 0.41, *BF*_01_ = 0.01). This means that participants actually spent more time processing prime stimuli than probe stimuli. This should work in favor of the binding mechanism since with increasing processing duration the likelihood of processing target and distractor sufficiently should increase (Shibuya & Bundesen, 1988). This should ensure that stimulus features are more likely available for binding mechanisms. Therefore, it is likely that not knowing the prime location in advance does affect the time participants spend looking at the prime display and this might generally enhance binding. Conversely, in the probe, participants spend less time searching the display because they know the target location in advance. Here it could be argued that less time spent on processing the probe display might reduce retrieval. Note, however, predictability of the target location in prime and probe is different per se but constant over all conditions it thus cannot explain our results concerning the modulation of DRB effects by grouping.

Our findings are intriguing on a theoretical level because they add to a growing body of literature that underlines that S-R binding can be separated into two separate processes (Laub et al., 2018; Mocke et al., 2020; Schmalbrock et al., 2021; Schmalbrock & Frings, 2021). Our results are also in line with previous conceptualizations of a rather automatic binding mechanism that almost indiscriminately binds stimuli in close spatial or temporal proximity with a response (Hommel, 2004). In Experiment 1, we observe no effect of our grouping manipulation on the binding effects, although we observed an impact of grouping on the prime RTs in general. This result suggests, that even separated stimuli became bound together nonetheless. In fact, the difference between binding effects in grouped and ungrouped trials was not significant (*p* = .147, one-sided) and the Bayes factor favors the null hypothesis (*BF*_01_ = 2.95, one-sided). Binding was clearly not modulated by our perceptual grouping modulation (but see Fournier & Gallimore, 2013, who found that grouping prime features does not lead to partial repetition costs).

In contrast, retrieval was reduced when the target and distractor were perceived as separate units. One possible explanation for this might be that because the distractor is perceived as separate, its processing begins later. Frings and Moeller (2012) showed that required and retrieved probe response rival for execution in a race-like competition (*horserace* account). Thus, if processing of the distractor stimulus starts later it will end up at a disadvantage. To clarify whether late selection explains the reduced DRB effect in the ungrouped condition of Experiment 2, we ran an additional trend analysis. Specifically, we looked at the DRB effects in the 25^th^, 50^th^, 75^th^, and 100^th^ percentile for the ungrouped condition of the RT distribution (i.e., DRB effects for fastest RTs to slowest RTs). However, the linear trend did not reached significance (*F*(1,89) = 1.63, *p* = .205, *η_p_*^2^ = .02).^6^ Late selection seems not to be the reason for the modulation. Alternatively, it is possible that the retrieval mechanism is rather flexible and is actively directed by contextual cues. This explanation would fit well with biased competition frameworks (e.g., Bundesen, 1990; Desimone & Duncan, 1995) where stimulus processing is directed by so-called *feature weights*. These weights determine whether a stimulus receives access to the capacity-limited processing resources and how much resources are distributed towards a stimulus – put simply, how important it is to process a stimulus. Importantly, feature weights can be increased or decreased by different variables (see Theeuwes & Failing, 2020 for a review). Feature weighting is claimed to also work in and through perceptual grouping (Vecera, 2000). For the present study, this could mean that feature weights of distractors (i.e., attention towards distractors) were reduced when they were presented as separate units to the target. This fits well with the findings that retrieval is sensitive to varying attention (Ihrke et al., 2011).

Yet, our findings stand in contrast to other gestalt manipulations that used other principles to modulate binding versus retrieval. Laub et al. (2018) used the grouping principle *similarity of color* (Wertheimer, 1923) as a modulator for binding and retrieval. In their first experiment, they presented a central target letter alongside distractor letters. Crucially, distractor letters could have a color that is more similar to the target color or less similar. In a second experiment, they used a similar setup where target and distractor were presented one after the other while target and distractor could have more or less similar colors. Importantly, they manipulated binding and retrieval separately but in the same trial (e.g., prime displays could be grouped while probe displays could be ungrouped, etc). They found that similarity in color enhances binding strength but dissimilarity of color actually enhances retrieval strength – yet, their study used a rather unusual design with targets and distractors not presented simultaneously but in succession, and separate prime/probe manipulations taking place in the same trial. Thus, their and our present study are hardly comparable because their way of manipulation makes it difficult to separate binding and retrieval modulation from each other, and the temporal dimension.

Building on previous work that modulated perceptual grouping in prime and probe displays and in line with recent theorizing of Hommel (2022), we suggest that perceptual grouping (here the principle of common region) modulates event file retrieval irrespective of binding processes while event file binding itself was not significantly modulated by grouping. Our results support the importance of recent theorizing that binding and retrieval are separate processes contributing to binding effects individually (Frings et al., 2020).

## Data accessibility statement

The data for Experiment 1 and 2 are both available at PsychArchives under *http://dx.doi.org/10.23668/psycharchives.5364*, and none of the experiments were preregistered.

## Figures and Tables

**Figure A1:**
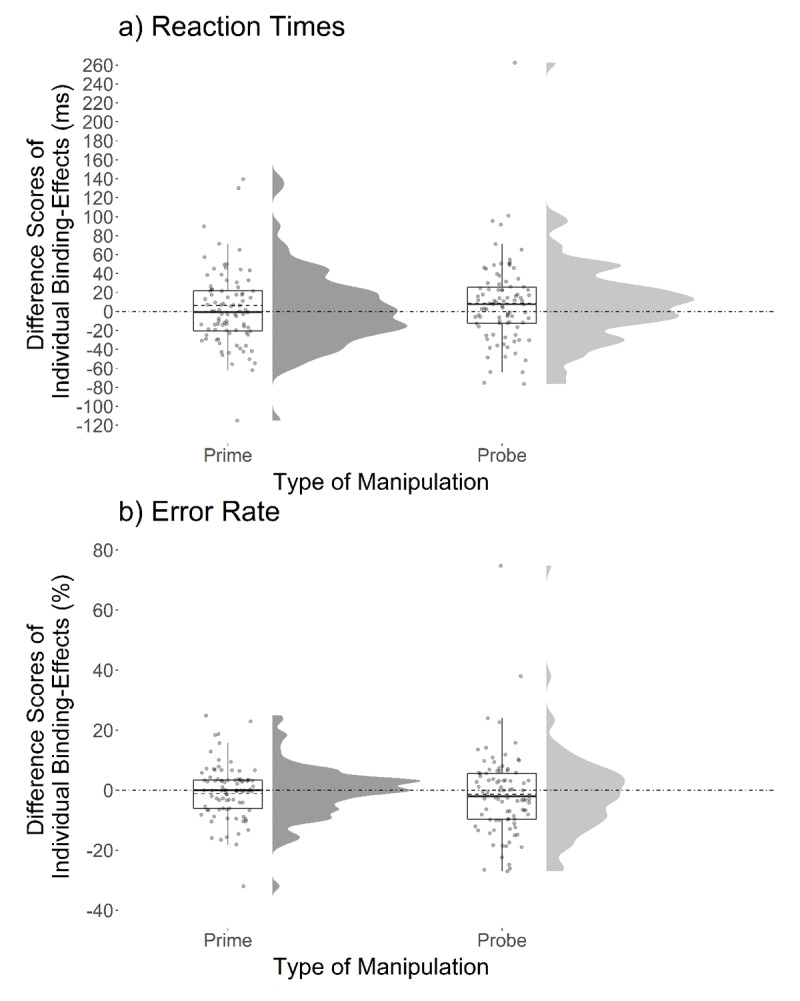
Difference Scores of Individual Binding Effects. Raincloud plots (Allen et al., 2019) for differences of individual binding effects (grouped – ungrouped) for a) Reaction Times and b) Error Rates in Experiment 1 (Prime) and Experiment 2 (Probe). The solid horizontal line in each boxplot represents the median of the distribution, the dashed line represents the mean of the distribution. Upper and lower whiskers extend to the largest/smallest value above/below the respective hinge but at most 1.5 times the interquartile range above and below the third and first quartiles (McGill et al., 1978).

## Footnotes

We use abbreviations for these four conditions. These abbreviations are derived from the two conditions Response Relation and Distractor Relation. That is, response change trials are abbreviated with RC and response repetition trials with RR. Distractor change trials are abbreviated with DC and distractor repetition trials with DR. Thus, the four conditions are: response change with distractor change – RCDC; response change with distractor repetition – RCDR; response repetition with distractor change – RRDC; response repetition with distractor repetition – RRDR.

Please note that in an initial calculation on sample size, we had an error and thus started to recruit/analyze data of 64 students first. To achieve sufficient power after we encountered this error, in accordance with the reviewers we decided to increase the sample size to 90 participants to achieve the initially intended power based on the expected effect size.

Note that this is just another, simpler way to present the two-way interaction between response relation and distractor relation. Comparing the DRB effects in the grouped condition with the ungrouped condition reveals this equivalence. When comparing the square-root of the relevant *F*-Value (i.e., the *t*-value) and the *p*-value of the ANOVA (see the **Supplementary Material**) to the *t*- and *p-*value of the *t*-test comparing DRB effects in the grouped vs. ungrouped condition. Both values are identical for both analyses.

Like in Experiment 1, we initially collected data of only 64 participants due to an erroneous calculation and added data after we encountered this error to reach the intended power.

Note that several publications showed that DRB effects cannot be alternatively explained via target retrieval alone. Even if targets always change from prime to probe DRB effects are still observed (Frings et al., 2007; Moeller et al., 2016). For example, Frings et al. (2007, Experiment) used a standard DRB paradigm and mapped four letters to a response key (there were two response keys, i.e., 8 target letters in total). For their analysis, they excluded all target repetition trials (i.e., identical targets in prime and probe). DRB effects still emerged – although prime and probe target were always two completely different letters. This indicates that distractors retrieve previous responses independent of target repetitions. The present study used a design in which target and response repetitions were confounded. Thus, it cannot be ruled out that target binding processes might also have contributed to our effects.

Mean binding effects for the ungrouped condition as a function of quantile: Quantile 25%: *M* = 3 ms, *SD* = 70; Quantile 50%: *M* = 9 ms, *SD* = 51; Quantile 75%: *M* = 10 ms, *SD* = 67; Quantile 100%: *M* = 30 ms, *SD* = 134. No other trend reached significance either, all *F*s < 2.00, all *p*s > .162.

## Appendix B

### RT ANOVAS for Experiment 1 and 2

Experimental conditions were compared using a repeated-measures analysis of variance (ANOVA) with type-III sums of square, using the ‘ezAnova’- function from the package ‘ez’ (Lawrence, 2016). We report two effect sizes for ANOVAs: *η_p_*^2^ for its widespread use in the literature, and *η_G_*^2^ as an appropriate measure of effect sizes in repeated measure designs (Bakeman, 2005).

**Table 1:** Results of the probe RT ANOVA for Experiment 1.

EFFECT	DEGREES OF FREEDOM	F-VALUE	P-VALUE	*η_G_* ^2^	*η_P_* ^2^

Response Relation	1/89	254.34	<.001	.09	.74

Distractor Relation	1/89	16.32	<.001	<.01	.15

Grouping	1/89	28.17	<.001	<.01	.24

Response Relation × Distractor Relation	1/89	41.34	<.001	<.01	.31

Response Relation × Grouping	1/89	1.74	.191	<.01	.02

Distractor Relation × Grouping	1/89	3.01	.086	<.01	.03

Response Relation × Distractor Relation × Grouping	1/89	1.11	.294	<.01	.01

**Table 2:** Results of the probe RT ANOVA for Experiment 2.

EFFECT	DEGREES OF FREEDOM	F-VALUE	P-VALUE	*η_G_* ^2^	*η_P_* ^2^

Response Relation	1/89	124.56	<.001	.05	.58

Distractor Relation	1/89	1.15	.286	<.01	.01

Grouping	1/89	5.93	.017	<.01	.06

Response Relation × Distractor Relation	1/89	28.75	<.001	<.01	.24

Response Relation × Grouping	1/89	3.15	.080	<.01	.03

Distractor Relation × Grouping	1/89	0.52	.471	<.01	<.01

Response Relation × Distractor Relation × Grouping	1/89	3.31	.072	<.01	.04
